# Preparation of a Sulfonated Carbonaceous Material from Lignosulfonate and Its Usefulness as an Esterification Catalyst

**DOI:** 10.3390/molecules18078168

**Published:** 2013-07-10

**Authors:** Duckhee Lee

**Affiliations:** The Division of Bionanochemistry, the College of Natural Sciences, the Wonkwang University, Iksan City, Chonbuk 570-749, Korea; E-Mail: dl202@wku.ac.kr; Tel.: +82-63-850-6225; Fax: +82-63-850-7312

**Keywords:** sulfonated carbonaceous material, lignosulfonate, solid acid catalyst

## Abstract

Sulfonated carbonaceous material useful as a solid acid catalyst was prepared from lignosulfonate, a waste of the paper-making industry sulfite pulping process, and characterized by ^13^C-NMR, FT-IR, TGA, SEM and elemental analysis, *etc*. The sulfonic acid group density and total density of all acid groups in the sulfonated carbonaceous material was determined by titration to be 1.24 mmol/g and 5.90 mmol/g, respectively. Its catalytic activity in the esterification of cyclohexanecarboxylic acid with anhydrous ethanol was shown to be comparable to that of the ionic exchange resin Amberlyst-15, when they were used in the same amount. In the meantime, the sulfonic acid group was found to be leached out by 26%–29% after it was exposed to hot water (95 °C) for 5 h. The catalytic usefulness of the prepared carbonaceous material was investigated by performing esterifications.

## 1. Introduction

Over the recent two decades or so carbon-based solid acids have received much attention in the fields of catalysis and organic synthesis [1,2,3,4,5,6,7,8,9,10,11,12,13,14]. Like other solid acids, they have several advantages such as readiness of separation from the reaction mixture, resulting reusability, and reduction in the amount of waste water generated in washing processes for removing the remaining acid from products, compared to homogenous catalysts such as *p*-toluenesulfonic acid or sulfuric acid when used in organic reactions. Among the carbon-based solid acids reported so far in the literature, sulfonated carbonaceous materials are most noteworthy since they are strong enough acids to be used as a catalyst for several types of organic reactions, thermally stable up to 150–200 °C, and are also prepared from cheap raw materials such as aromatic hydrocarbons, carbohydrates, and sulfuric acid. Since first reported by Hara *et al*. [15], several research groups have shown that amorphous carbon bearing SO_3_H groups could be prepared by treating various types of organic compounds with conc. sulfuric acid [16,17,18,19,20,21,22,23,24,25]. In this preparation, a series of reactions like elimination, rearrangement of the carbon back bone, gas evolution, condensation, and sulfonation, *etc*., were known to occur in one pot to give carbonaceous compounds with graphene-like structures containing SO_3_H, OH, and CO_2_H groups on the edge of their sheet-structure molecules. Here a new preparative method for such catalysts using as a raw material lignosulfonate, which is a by-product of the sulfite pulping process of the paper-making industry, is presented, together with some catalytic characteristics of the product. As a starting material, lignosulfonate is an interesting compound because it already has a sulfonic acid group on the sp^3^ carbon of the molecule and an activated aromatic ring available for electrophilic sulfonation. Therefore it was chosen as a starting material with a hope that some catalyst characteristics such as density of SO_3_H groups and resistance to hydrolysis might be improved.

## 2. Results and Discussion

### 2.1. Material Synthesis and Acid Density, BET Surface Area

**Scheme 1:**
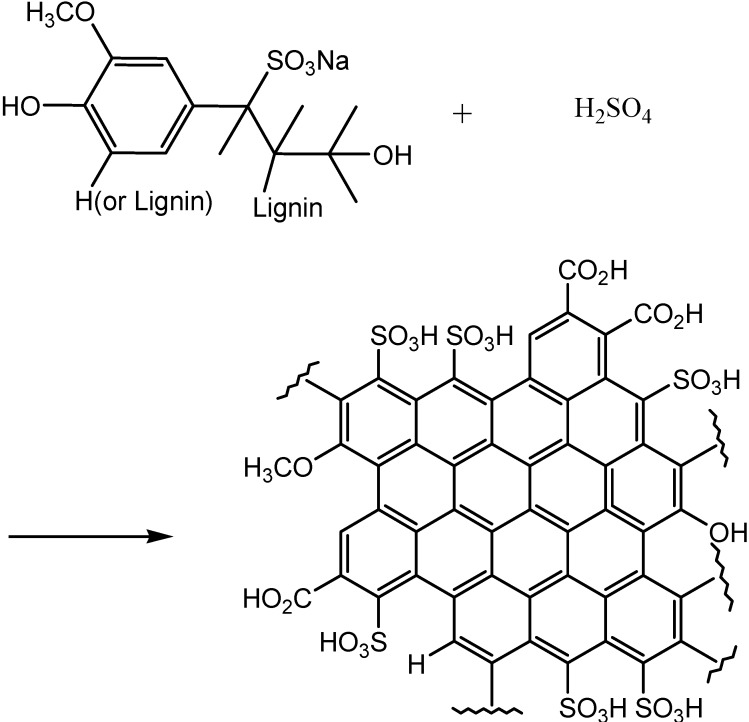
Preparation of the sulfonated carbonaceous material.

The catalyst was prepared from the lignosulfonate according to Scheme 1. Conc. sulfuric acid and fuming sulfuric acid were used as a sulfonating agent in the reactions. The same type of reaction was carried out with glycerol to obtain a standard material for comparison.

After the initial exothermic reaction settled down, the reaction product was filtered and thoroughly washed with deionized water until no sulfate ion was detected by a test using aqueous barium chloride solution. 

The reaction conditions and acid density values obtained by titration on the products are summarized in Table 1 together with the elemental analysis data and BET surface area data. The surface areas of the synthesized carbonaceous materials were small, 2.18–3.19 m^2^/g and not much different from that of the material made of glycerol, 1.79 m^2^/g. The low surface area may be attributable to the non-porous structure of the materials. The density of SO_3_H groups in the material was 1.24 mmol/g, indicating a much lower value than that of the commercial ion exchange resin , Amberlyst-15 while the total acidity expressed as a sum of all acidic groups such as phenolic OH, CO_2_H, and SO_3_H was found to be similar to that of the well-known ion exchange resin Amberlyst-15. The higher value of the total acidity than the density of SO_3_H group can be interpreted as due to the presence of many acidic groups like phenolic OH or CO_2_H groups in addition to SO_3_H groups in the materials. It is interesting that the sulfonated carbonaceous material prepared with fuming sulfuric acid (run 3) has a higher total acidity value than the other samples. This can be attributed to the higher oxidizing ability of the fuming sulfuric acid, compared with normal sulfuric acid.

**Table 1:** The acid density and surface area of the prepared carbonaceous material with SO_3_H groups.

Samples		N_2_ adsorption (Surface area)	E.A	T. A (mmol H^+^/g)	SO_3_H (mmol/g)	CO_2_H (mmol/g)	OH (mmol/g)
Run	(Raw materials)						
1	Gly(100%), Sul	1.79 m^2^/g	^4^N.A	4.86	1.24	3.66	0
2	^1)^Lig(100%), Sul	3.19 m^2^/g	CH_0.72_O_0.53_S_0.017_	4.64	0.96	3.36	0.32
3	^2)^Lig(100%),Sul(fum)	2.18 m^2^/g	CH_0.59_O_0.26_S_0.015_	5.90	1.24	3.66	1.04
4	^3)^Amberlyst-15	45 m^2^/g	^4)^N.A	4.66	4.60	N.A	N.A

Abbreviations: Gly: Glycerol, Lig: Sodium Lignosulfonate, Sul: 96% sulfuric acid, Sul(fum): sulfuric acid (20% fuming) E.A: Elemental analysis, T. A: Total acidity, SO_3_H, CO_2_H, and OH represent their density respectively. 1) Reaction temperature: 150–175 °C, Reaction time: 0.5 h; 2) Two continuous reactions: the first reaction; Reaction temperature: 150–175 °C, Reaction time: 50 min, and then followed by the second reaction; reaction temperature: 150–175 °C, reaction time: 12 hr, sulfonating agent: 20% fuming H_2_SO_4_; 3) The data on Amberlyst-15 were adapted from reference [11]; 4) N.A: not available.

### 2.2. Spectroscopic Studies by IR and Solid-state ^13^C-NMR

**Figure 1:**
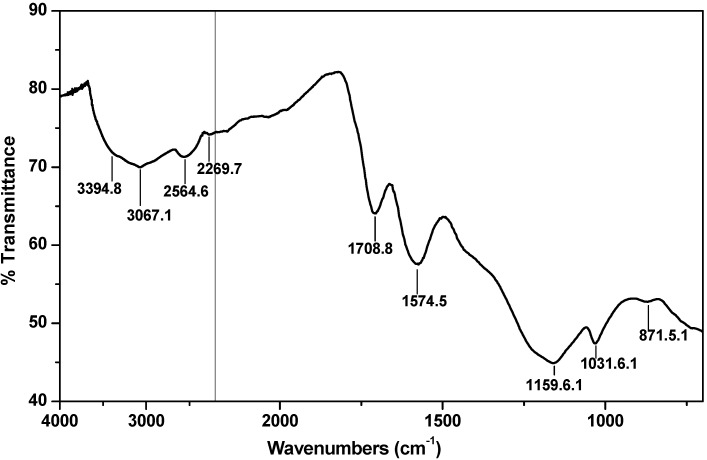
FT-IR spectrum of the prepared carbonaceous material made from lignosulfonate.

The FT-IR spectrum of the SO_3_H group-bearing carbonaceous material is shown in the Figure 1. The peaks at 1709 cm^−1^ and at 1159 cm^−1^ are due to the presence of CO_2_H and C-O functional groups, respectively. The peaks due to the SO_3_H group are seen at 1032 cm^−1^ and at 872 cm^−1^. The peaks attributable to aliphatic C-H bond, aromatic C-H bond, O-H bond in a carboxylic acid group, and O-H bond in a phenol group are seen as a broad peak between 3600 cm^−1^ and 2400 cm^−1^.

**Figure 2:**
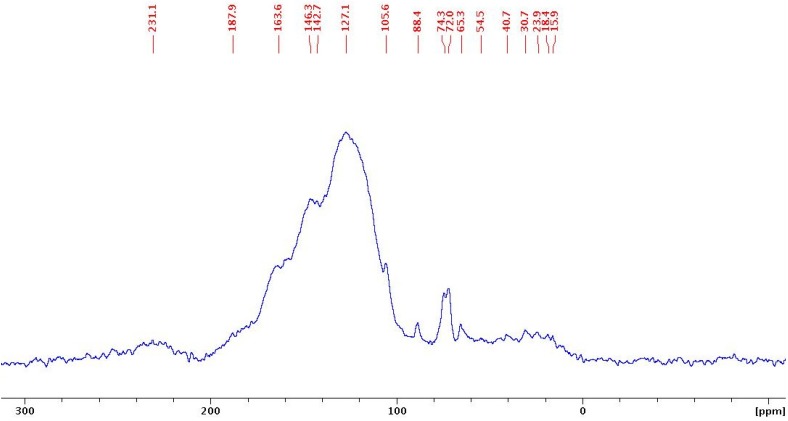
^13^C CP/MASNMR spectrum of the sulfonated carbon obtained with the magic angle spinning technique.

In the meantime a ^13^C-MAS-NMR experiment was carried out to obtain clues regarding the structure of the sulfonated carbon. The spectrum is shown below in Figure 2.

The peaks at around 127, 146, and 188 ppm can be assigned to polycyclic aromatic carbon atoms, phenolic OH-bearing carbons, and carbonyl carbon atoms of carboxylic acid groups, respectively, as reported previously in the literature [26]. The resonance peak due to SO_3_H group-bearing aromatic carbons is seen as a little obscure peak around 139 ppm in this spectrum because of the overlapping peaks due to phenolic OH-bearing carbons and polycyclic aromatic carbons.

### 2.3. Hydrolytic Stability Studies

The hydrolytic stability of the sulfonated carbonaceous material was studied by exposing it to hot (95 °C) water for 5 h. The amount of sulfonic acid groups leached during the exposure was determined to be 29% of the initial sulfonic groups in the material by titration with standardized 0.1 N sodium hydroxide solution after filtering the solid materials. The density of the sulfonic groups in the collected solid material also turned out to be decreased by 25% by exchanging protons in the sulfonic group with 1 M sodium chloride solution followed by titration with standardized 0.1 N hydrochloric acid solution. This leaching of the sulfonic acid groups implies that a considerable portion of sulfonic groups in the molecule are linked to sp^2^ hydridized carbon atoms just as in the case of benzenesulfonic acid. The desulfonation of benzenesulfonic acid under hydrolytic conditions is well-known reaction. Therefore the leaching can be ascribed to the desulfonation occurring at the sp^2^ carbon atoms containing a sulfonic acid group. When the sulfonated carbonaceous material is used as a catalyst, catalysis by the leached sulfuric acid might be important under harsh reaction conditions in which desulfonation is facilitated.

### 2.4. Thermal Stability Studies by TGA

**Figure 3:**
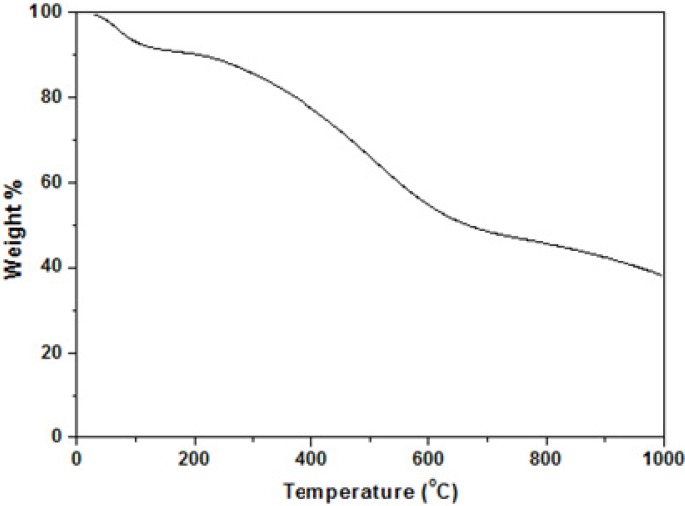
Thermogram depicting weight loss due to functional group decomposition.

The thermal stability of the sulfonated carbonaceous material was examined by thermogravimetric analysis (TGA) under a nitrogen gas atmosphere. As shown in Figure 3, the material started to lose weight from 50 °C. This can be attributed to the loss of water absorbed on the surface of the carbonaceous material. The second stage of the weight loss appeared around 230 °C can be interpreted as due to the decomposition of the SO_3_H groups. This TGA result resembles that of the sulfonated carbon made by sulfonation of aromatic hydrocarbons such as naphthalene with concentrated sulfuric acid [15]. There is about 40% of the material weight still left at 1,000 °C. The remaining material is thought to be graphene-like carbon bodies. This TG profile supports the structure of the sulfonated carbon depicted in Scheme 1.

### 2.5. Catalytic Activity Studies

**Scheme 2:**
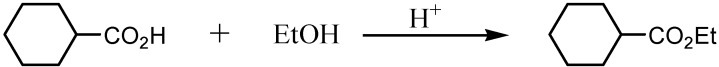
Synthesis of Ethyl Cyclohexanecarboxylate

Catalytic p**r**operties of the sulfonated carbonaceous material was investigated by carring out acid-catalyzed esterifications of cyclohexanecarboxylic acid with ethanol (Scheme 2). 

The reaction was repeated with different catalysts in the same conditions (same temperature and same reaction time) and their results are summarized in Table 2. The same equivalents of catalysts measured as as a proton (0.372 mmol proton) in strongly acidic groups used in each reactions except for run 1 and 3. 

**Table 2:** Yields and reaction conditions of the esterification of cyclohexanecarboxylic acid with ethanol

Reaction Run	Substrate(g)	^2)^Alcohol(g) 10eq.	Catalyst(g)	Temperature(°C)/Reaction Time(hr)	^7)^Yields(%)
1	^1)^CHCA(1.28)	EtOH(4.6)	c-H_2_SO_4_(0.1)	(76)/(7)	98
2	CHCA(1.28)	EtOH(4.6)	c-H_2_SO_4_(0.0182)	(76)/(23)	88
3	CHCA(1.28)	EtOH(4.6)	^3)^Amberlyst-15(0.3)	(76)/(23)	83
4	CHCA(1.28)	EtOH(4.6)	^4)^SC-I(0.3)	(76)/(23)	85
5	CHCA(1.28)	EtOH(4.6)	^5)^SC-I(0.3)	(76)/(23)	84
6	CHCA(1.28)	EtOH(4.6)	^6)^SC-II(0.3)	(76)/(23)	92
7	Levulinic Acid(2.90)	EtOH(11.5)	Amberlyst-15(0.3)	(76)/(23)	75
8	Levulinic Acid(2.90)	EtOH(11.5)	SC-I(0.3)	(76)/(23)	67
9	Levulinic Acid(2.90)	EtOH(11.5)	^5)^SC-I(0.3)	(76)/(23)	51

1) CHCA: cyclohexanecarboxylic acid; 2) anhydrous EtOH, 3) Dried prior to use at 110°C for 13 hrs; 4) SC-I (sulfonated carbon prepared from lignosulfonate by single sulfonation); 5) Reused after 1^st^ use; 6) SC-II (sulfonated carbon prepared from glycerol by sulfonation); 7) Isolated yields of the ester products based on the amount of the carboxylic acid used.

High yields were obtained in the most of the cases despite the fact no any entrainer or Dean-Stark trap were used to remove water formed in the equilibrium reaction. This result can be understood on the basis of the well-known Le Chatelier’s principle, *i.e*., using one of the reactants (EtOH) in excess amount (10 equiv.) makes the reaction equilibrium shift to the forward reaction. 

As shown in runs 2, 3, 4, 6, the sulfonated carbon prepared from lignosulfonate gave slightly lower yields than sulfuric acid, but a little better yield than the case of a well-known ionic exchange resin, Amberlyst-15, which has a much higher density of SO_3_H groups than that of the sulfonated carbon. This means that the sulfonated carbon catalysts have better catalytic ability than the Amberlyst-15 in spite of their relatively low surface area and low SO_3_H group density. This difference in catalytic reactivity can be explained in terms of accessibility of reactants to the catalytic sites. From the spectroscopic data on their structure it is reasoned that the sulfonated carbon catalysts consist of two parts, namely a hydrophobic graphene-like domain and hydrophilic edges which are linked to SO_3_H, OH, and CO_2_H groups. Undoubtedly the catalytic ability of the carbon catalysts comes from these acidic groups, especially the SO_3_H groups. Reactants can get access to all of the catalytic sites freely in the case of the sulfonated carbon catalysts while they can only reach some of the SO_3_H groups in the case of the Amberlyst-15. This is probably because some SO_3_H groups that are located inside of the resin are not accessible due to steric effects. In addition to the steric reasons, hydrophobic interactions between the graphene-like domain and the non-polar part of reactants, the cyclohexyl group in this case, is also likely to contribute to some extent to the enhanced catalytic efficiency of the sulfonated carbon catalysts. 

In the meantime the comparable yields of run 5 and run 4 indicate that the catalyst can be used repeatedly in this reaction. It is interesting that the sulfonated carbon made from glycerol gave a slightly better yield than the case of lignosulfonate-orginated sulfonated carbon (run 6) although they showed the same degree of sulfonic acid (SO_3_H) group density in the analysis by titration. The above results strongly suggest again that the reaction rate in this reaction is strongly influenced not only by the catalytic site density, but also by other properties of the catalyst such as adsorption ability or accessibility to substrates. These results are similar to high activity of a sulfonated charcoal catalyst reported by Yurui in the esterification of polyhydric alcohols [1]. 

**Scheme 3:**
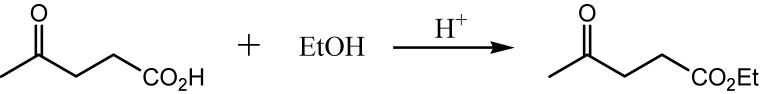
Synthesis of ethyl levulinate.

Ethyl levulinate is a biofuel fuel-additive, which can be added up to 5 wt % as a diesel-miscible biofuel directly in regular diesel car engines [27]. Several homogeneous mineral acids such as H_2_SO_4_, H_3_PO_4_, and HCl were used to prepare this ester in the previous research [28,29,30,31] (Scheme 3).

Since even trace amounts of acid can seriously affect such engines, in its production it is highly desirable to use a solid acid catalyst which is easier to separate from the reaction mixture after use, use repeatedly and has no need for neutralization. As shown the above Table 2 (run 8), the catalytic activity of the sulfonated carbon made of lignosulfonate was found to be good enough to be used for production of such an industrially valuable compound although it showed slightly less activity than Amberlyst-15. However a somewhat decreased yield (run 8) was obtained with the recovered catalyst. This was an unexpected result considering the fact that almost the same yields were obtained in the previous cases (runs 4 and 5). This might be because the catalyst underwent more effective desulfonation in a more polar reaction medium consisting of ethanol and levulinic acid than in a reaction medium consisting of cyclohexanecarboxylic acid and ethanol.

## 3. Experimental

### 3.1. Instruments and Reagents

The solid state ^13^C CP/MAS-nmr data was acquired at Bruker AVANCE II^+^ 400MHz NMR system (located at the KBSI Daegu Center, Korea). ^1^H-NMR and ^13^C-NMR spectra were recorded on a Jeol 500 MHz NMR spectrometer. The elemental analysis was performed using a Perkin Elmer 2400 series II elemental analyzer. The carbon, hydrogen, sulfur contents were determined directly while the oxygen content was calculated by difference. Thermogravimetric analysis (TGA) was performed using a GA Q50 thermogravimetric analyzer, (TA Instruments Inc., New Castle, DE, USA). The thermal stability of functional groups introduced during sulfonation was investigated by heating the samples at 10 °C/min to 1,000 °C under an inert atmosphere (N_2_ flow). The FT-IR spectral data were obtained in reflectance mode using an is5/FT-IR spectrometer (Thermo Fisher Scientific, Waltham, MA, USA). The surface images of the catalysts were obtained using a field emission scanning electron microscope (JSM-6701F/JEOL, Tokyo, Japan). The BET surface areas of the catalysts were measured using a BET Surface Area and Porosimetry Analyzer (model: ASAP 2020, Micromeritics Instruments, Norcross, GA, USA). The samples were pre-treated at 200 °C for 4 h prior to measurements. Sufuric acid, fuming sulfuric acid, glycerol, cyclohexanecarboxylic acid, and levulinic acid were purchased from Junsei (Tokyo, Japan), Sigma-Aldrich Korea(Seoul, Korea), and Daejung Chemicals & Metals Co. Ltd (Shiheung, Korea), respectively. All reagents were used as received without purification.

### 3.2. Synthesis

#### 3.2.1. Preparation of the Catalysts

##### 3.2.1.1. Preparation of the Catalyst from Sodium Lignosulfonate

Sodium Lignosulfonate (Mwt. ≈ 7,000, 20 g) was slowly added with stirring in several portions to a 300 mL of flask containing conc. sulfuric acid (140 g) over 30 min. so that the reaction temperature was kept below 200 °C. A lot of gas evolution was observed during stirring and the reaction mixture became black and insoluble. The reaction mixture was stirred at 150–175 °C for a further 30 min. After isolating from soluble materials by filtration, the precipitated material was pulverized in a mortar and then thoroughly washed with hot deionized water many times (at least more than 15 times!) until no sulfate ion was detected by an aqueous BaCl_2_ solution test. The black solid was dried at 110 °C overnight (13 h) to give 10.96 g of the pre-sulfonated carbon(SC-I). Elemental analysis: CH_0.72_O_0.53_S_0.017_. This pre-sulfonated carbon was sulfonated once more with fuming sulfuric acid (20%, 150 g) at 150–175 °C for 12 h. After filtration, washing, and drying, 7.0 g of the sulfonated carbon (SC-II) was obtained. Elemental analysis: CH_0.59_O_0.26_S_0.015_. FT-IR(film, cm^−1^): 3066 (aromatic C-H), 1714 (carbonyl group in carboxylic acid), 1575 (C=C in polyaromatic ring), 1150 (aromatic C-O), 1029(S=O in SO_3_H).

##### 3.2.1.2. Sulfonated Carbon II(SC-II)

Preparation of the catalyst from glycerol(for comparison). Glycerol (25g) was added dropwise to a flask containing conc. sulfuric acid (178 g) at 140 °C over 1 h so that the reaction temperature was kept below 200 °C. After exothermic reaction occurred, which subsidized as the reaction mixture was stirred at 180–200 °C for a further 1 h. After cooling down to room temperature and filtering, washing with deionized water, and drying as mentioned before, 5.72 g of the sulfonated carbon was obtained. SO_3_H density: 1.24 mmol/g, total acidity: 4.86 mmol/g. FT-IR (film, cm^−1^): 1705 (carbonyl group in carboxylic acid), 1575 (C=C in polyaromatic ring), 1155 (aromatic C-O), 1029 (S=O in SO_3_H), 897.

#### 3.2.2. Determination of the Acidity

The density of SO_3_H groups was determined by titration following the method reported in the literature [32]. Aqueous 1 N sodium chloride solution (50 mL) and the sulfonated carbon (0.5 g) were mixed in a flask and stirred at room temperature for 4 h. The solution was filtered through a short Celite pad to give clear solution. A 25-mL aliquot of the solution was taken and titrated with standardized 0.1 N sodium hydroxide solution using phenolphthalein indicator. The density of SO_3_H group (*N* mmol/g) was calculated as follows:

*N_SO3H_* (*m*Eq/g) = The volume of NaOH used(mL) per gram of the sulfonated carbon × 1 N (the normality of the NaOH solution).

The total acidity of the sulfonated carbons is expressed as a sum of the contributions from all of the acidic groups such as SO_3_H, OH, CO_2_H, *etc*. For measuring the total acidity, 0.1 N NaOH solution (100 mL, excess) and the sulfonated carbon (0.5 g) were mixed in a beaker. The mixture was shaken for 4 h to ensure that the solution had come to equilibrium. The solution was back titrated with standard 0.1 N HCl solution using a pH meter. The total acidity(*N_total_*) of the sulfonated carbon was calculated as follows:

*N_total_* (*m*Eq/g) = The volume of NaOH used(mL) initially per gram of the sulfonated carbon × 0.1 N (the normality of the NaOH solution) – the volume of HCl used × 0.1 N (the normality of the NaOH solution).

For determining the total amount of M-SO_3_H and M-CO_2_H, a literature method [14] was slightly modified. In this method a pH meter was used instead of a pH indicator because the solution to be titrated was dark brown in colour. The catalyst (0.1 g) was put into 0.08 M NaHCO_3_ solution (25 mL). The solution was sonicated for 2 h and filtered through a Celite pad. The Celite pad was washed with a small amount of deionized water (5 mL). At the beginning process of the titration the amount of NaHCO_3_ solution consumed should be equal to the total amount of M-_SO3H_ and M-_CO2H_ present in the catalyst. The filtrate was titrated with 0.1 N HCl using a pH meter (at an equivalent point pH = 3.90) and the unconsumed amount of NaHCO_3_ solution was calculated from the data. The amount of M-phenolic OH was determined by substracting (the sum of M-_SO3H_ and M-_CO2H_) from the total of M-_SO3H_, M-_CO2H_ and M-_OH_).

#### 3.2.3. Synthesis of the Esters

##### 3.2.3.1. Ethyl cyclohexanecarboxylate

Cyclohexanecarboxylic acid (1.28 g, 0.1 mmol), anhydrous ethyl alcohol (4.6 g, 1.0 mmol), and the catalyst (300 mg) were mixed in a 30 mL flask at °C and stirred at the same temperature for 23 h. After the reaction mixture was cooled down to room temperature it was filtered through Celite. The ethyl alcohol was evaporated under reduced pressure to give an oil that was diluted with diethyl ether (50 mL) and the solution was washed twice with deionized water. The ether layer was separated and dried over anhydrous magnesium sulfate. After removal of insoluble materials by filtration, the ether solution was evaporated to give the ethyl cyclohexanecarboxylate. When the sulfonated carbons (300 mg), Amberlyst-15 (300 mg), and conc, sulfuric acid (100 mg), conc, sulfuric acid (18.2 mg), were used as a catalyst in the reaction, the yields of the ester in each case were 92% (SC-II), 85% (SC-I), 84% (recovered SC-I), 83% (Amberlyst-15), 98% (H_2_SO_4_, 100 mg), and 88% (H_2_SO_4_, 18.2 mg), respectively. ^1^H-NMR data (CDCl_3_, δ): 4.08 (2H, q), 2.23 (1H, tt), 1.85 (2H, d), 1.70 (2H, d), 1.60 (1H, d), 1.39 (2H, q), 1.28–1.14 (6H, m); ^13^C-NMR data (CDCl_3_, δ): 176.3, 60.1, 43.3, 29.1, 25.8, 25.5, 14.3.

##### 3.2.3.2. Ethyl levulinate

Levulinic acid (5.81g, 50 mmol), anhydrous ethyl alcohol (4.61 g, 500 mmol), and the catalyst (500 mg) were added to a flask equipped with a thermometer and a reflux condenser. The reaction mixture was heated to 77 °C and then stirred at the same temperature for 23 h. After cooling down to room temperature, the solution was filtered through a Celite layer. The Celite layer was washed with ethyl alcohol (15 mL × 2) and the combined alcohol solution was evaporated to give an oil. The oil was diluted with diethyl ether (40 mL) and the solution was washed with deionized water (30 mL × 2). The ether layer was separated and dried with magnesium sulfate. After removing the magnesium sulfate by filtration, the ether layer was evaporated to give the ethyl levulinate. When the sulfonated carbons, and Amberlyst-15 were used as a catalyst in the reaction, the yields of the ester in each case were 75% (Amberlyst-15), 67% (SC-I), and 51% (SC-I recovered after 1^st^ use), respectively. ^1^H-NMR data (CDCl_3_, δ): 4.07 (2H, q), 2.70 (1H, t), 2.51 (2H, t), 2.14 (3H, s), 1.19 (3H, t); ^13^C-NMR data (CDCl_3_, δ): 206.9, 172.9, 60.7, 38.0, 29.9, 28.0, 14.2.

#### 3.2.4. Stability Test of the Sulfonated Carbons under Hydrolytic Condition

The sulfonated carbon (SC-I, density of SO_3_H: 1.24 mmol/g, 0.5 g) was dispersed in deionized water (50 mL) and the solution was stirred at 95 °C for 5 h. After removing the catalyst by filtration, the filtrate was titrated using a pH meter and standardized 0.1 N sodium hydroxide solution. From the amount of the sodium hydroxide solution consumed (1.8 mL), the amount of sulfonic acid groups leached from the sulfonated carbon for the period was calculated to be 29% of the initial sulfonic acid groups in the sulfonated carbon. The amount of leached SO_3_H groups was checked once more with the catalyst collected on the filter as follows. The collected sulfonated carbon on the filter was contacted with 1.0 N sodium hydroxide solution (50 mL) at room temperature for 4 hrs. After removal of the solid by filtration, 25 mL of the filtrate was taken in a beaker and titrated using standardized 0.1 N hydrochloric acid solution and a pH meter. From the amount of hydrochloric acid consumed (2.3 mL), the density of sulfonic acid groups in the recovered sulfonated carbon was calculated to be 0.92 mmol/g.

## 4. Conclusions

A sulfonated carbon, which can be used as a solid acid catalyst for esterifications, was prepared from sulfuric acid and sodium lignosulfonate a waste material by-product of the sulfite pulping process in paper-making industry. The carbon material was found to possess 1.24 mmol/g of sulfonic acid groups and 4.6 mmol/g of total acidity. The sulfonated carbon was also shown to be stable up to around 230 °C. However only moderate stability was observed when it was exposed to hydrolytic conditions, *i.e.*, a 26%–29% decrease in the density of SO_3_H groups after 5 h of exposure to hot water (95 °C) was observed. In the meantime, it is well-known that SO_3_H group-bearing materials like Amberlyst-15 can be used as a cation exchanger, therefore the sulfonated carbon prepared in this study is likely to find utility as a packing material in ion chromatography using such cation exchangers or in column chromatography for the separation of organic compounds.
